# Characterization of differential gene expression of broiler chicken to thermal stress in discrete developmental stages

**DOI:** 10.1080/19768354.2022.2059566

**Published:** 2022-04-05

**Authors:** Hyun Seung Kim, Jimin Kim, Jaemin Kim, Yang Ho Choi

**Affiliations:** a Division of Applied Life Science (B. K.21 Plus) and Gyeongsang National University; b Institute of Agriculture and Life Sciences, Gyeongsang National University, Jinju, Korea; c Department of Animal Science, Gyeongsang National University, Jinju, Republic of Korea

**Keywords:** Microarray, differentially expressed genes, heat stress, arbor acres broilers

## Abstract

Prolonged exposure to high temperatures is linked to a range of physiological responses in broiler chickens including reduced disease resistance, low growth rate, and high mortality rate. In this study, we investigated the effect of heat stress on gene expression levels in 4-week-old and 6-week-old chickens each exposed to environments conditioned at thermoneutral (21 °C) and high (32 °C) temperatures. The analysis of differentially expressed genes (DEGs) using microarray revealed that genes underlying reactive oxygen species (ROS) production, cell nutrient intake, glucose metabolism, and circadian rhythm were differentially regulated in association with heat stress. We also found that the deviation in expression levels across the transcriptome in response to heat stress was significantly stronger (*P*< 2.2×10^−16^) in 6-week-olds compared to younger chickens. We finally observed a significant trend (*r* = 0.78, *P*< 2.2×10^−16^) that genes with a higher estimate of expression in the microarray were more likely to have a higher expression level in RNA-sequencing. Together, our findings provide comprehensive insights into the physiology involved in stress responses at varying developmental stages, which may facilitate chicken breeding to maximize their productivity under adverse conditions.

## Introduction

Chicken (*Gallus gallus domestica*) provide an irreplaceable source of food, but a major constraint on their productivity is heat stress, which leads to stunted growth, increased rate of mortality, decreased reproduction, and reduced meat quality (Irshad et al. 2013). Especially, a rapid growth rate of broiler chickens causes more extensive and irreversible molecular and physiological changes under harsh environmental stressors including high and low-temperature extremes (Giloh et al. 2012; Nascimento et al. 2017).

Heat exposure alters the metabolic signatures and stimulates oxidative damage to broiler skeletal muscles in avian species by upregulating the levels of enzymes that govern β-oxidation, TCA cycle, and fatty acid transport (Mujahid et al. 2007). It is also known to increase the level of malondialdehyde (MDA) which is an indicator of lipid peroxidation (Wang et al. 2009). The cytotoxicity induced by heat, excessive inflammation, and inability to increase the expression of heat shock proteins (HSPs) altogether lead to the pathogenesis of heat injury and associated tissue damage (Rowell L 1983). The heat exposure also causes ferroptosis-like death in mammary gland tissues of goat and affects breastfeeding and productivity (Liu et al. 2021). To fully understand the molecular anatomy of heat-associated morbidity and mortality, several studies have attempted the analyses at the transcriptome-wide level (Jung et al. 2020). Srikanth et al. recently attempted to investigate transcriptomic responses in low and highland chickens experienced thermal challenges (Srikanth et al. 2019). Jastrebski et al. integrated transcriptome and metabolome data to examine the hepatic responses of chickens under chronic heat stress (Jastrebski et al. 2017). Nevertheless, it is still unclear how thermoregulatory mechanisms may vary across different developmental stages especially in a rapidly growing broiler chicken. Here, we generated the whole genome-wide expression levels in breast muscle of 4-week-old and 7-week-old broiler chickens exposed at the environments conditioned at moderate (21 °C) and high (32 °C) temperatures.

## Materials and Methods

### Ethics approval

The animal study was approved by the Animal Care and Use Committee of Gyeongsang National University (GAR-110818-X0035).

### Heat stress challenge experiment

In summary, this study involved chickens from two developmental stages; 4 and 6-week-old broiler chickens (4W and 6W, respectively), each comprised a treatment group exposed to the environments conditioned at 32 °C (4WH and 6WH, n = 3 each) and a control group at 21 °C (4WC and 6WC, n = 3 each). The temperatures under thermoneutral and hyperthermic conditions were derived from previous studies (Lin et al. 2006; Yoon et al. 2014).

A total of 240 one-day-old broiler chicks were procured and raised under a light cycle of 23 h of light and 1 h of dark. Birds were provided *ad libitum* access to water and fed the same diet. The temperature was decreased by 1 ∼ 2°C every two days thereafter until the temperature reached 21 ± 1°C on the day 21 post-hatch. The 4-week (n = 120) and 6-week-old (n = 120) chicks were each further raised in two separate houses: thermoneutral (21 °C, control group) and heat-stressed (32 °C, treatment group) conditions. For the treatment group, the temperature was gradually increased by 2 °C per hour to reach 32 °C. After one day of high and moderate temperature exposure, three birds from each experimental group (4WC, 4WH, 6WC, and 6WH) were randomly selected and euthanized. A total of twelve breast muscle tissue samples were collected and stored at −80 °C for analysis. Microarray analysis was performed using Affymetrix GeneChip chicken genome arrays containing 38,535 probes (Affymetrix, Santa Clara, CA).

### Analysis of differentially expressed genes

R (ver.4.1.0) packages (Team 2013) ‘limma’ and ‘affy’ were used to analyze the gene expression profile data by quantifying the microarray fluorescence intensities. The probes with fold change ≥1.5 and FDR < 0.05, were considered significant. After filtering the cases where multiple probes matched with one gene and one probe matched with multiple genes, the differentially expressed genes (DEG) were defined for each heat stress challenge experiment.

### Gene set enrichment analysis

DAVID version 6.8 (http://david.abcc.ncifcrf.gov/) (Huang et al. 2009) tool was used to identify significant over-representation of genes with particular functional categories such as Gene Ontology (Ashburner et al. 2000) and KEGG Pathway (Kanehisa and Goto 2000). The *P*-value of 0.05 was used as the criterion for the statistical significance.

### RNA-sequencing data and expression

The 17 RNA-sequencing (RNA-seq) data for the breast muscle and 86 RNA-seq data for various tissues of the chicken were obtained from NCBI (National Center for Biotechnology Information) (https://www.ncbi.nlm.nih.gov/) (Wheeler et al. 2007) (Supplementary Table S1).

The RNA-seq data was treated by Trimmomatic (ver. 0.39) (Bolger et al. 2014) to filter low-quality reads and then RSEM (ver. 1.3.1) (Li and Dewey 2011) software to quantify and estimate the expression level of each gene (transcript per million, TPM) with Galgal 4.85 used as the reference genome. The correlation coefficient was estimated between the microarray and the TPM of RNA-seq data The gene expression levels of both platforms were log-transformed before comparison.

### Figure visualization and statistical analyses

Boxplots and heatmap plots were generated using ‘ggpubr,’ ‘gplot2,’ ‘gplot’ and 'wesanderson’ packages of R. We conducted a principal component analysis between each sample and created a plot using the R package ‘ggfortify’

## Results & discussion

### Experimental design and overall expression patterns

**Figure 1.**
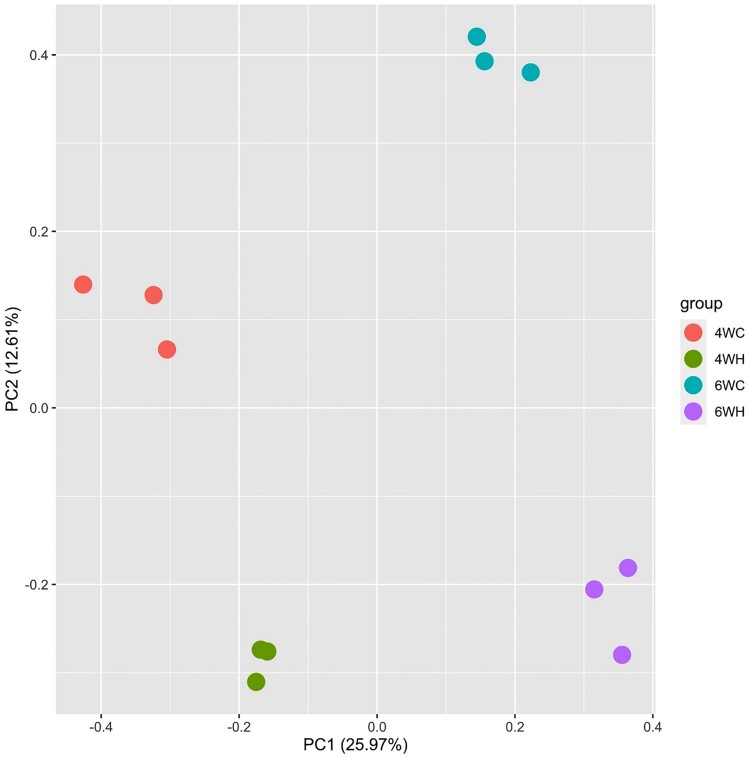
Principal component analysis showing PC1 against PC2 of all twelve samples comprising 4 experimental groups: 4-week-old control (4WC, n = 3), 4-week-old high temperature (4WH, n = 3), 6-week-old control (6WC, n = 3), and 6-week-old high temperature (6WH, n = 3).

We generated the microarray-based transcriptome data of breast muscle from 4-week and 6-week-old chickens at moderate and high-temperature groups (4WH, 4WC, 6WH, and 6WC) (Materials and Methods). The 4W and 6W represent two broiler growth phases of different growth rates, and the bodyweight of the later period was significantly higher (Roush and Wideman Jr 2000; Mountzouris et al. 2010). We performed principal component analysis (PCA) of the genome-wide expression profiles (Figure 1). The analysis ignored group membership but revealed clear experiment distinction as samples from the same experimental group clustered together. The first PC (25.97%) separated developmental stages (4W versus 6W) and PC2 (12.61%) further differentiated expression profiles in the treatment and control group (high and moderate temperatures).

**Figure 2.**
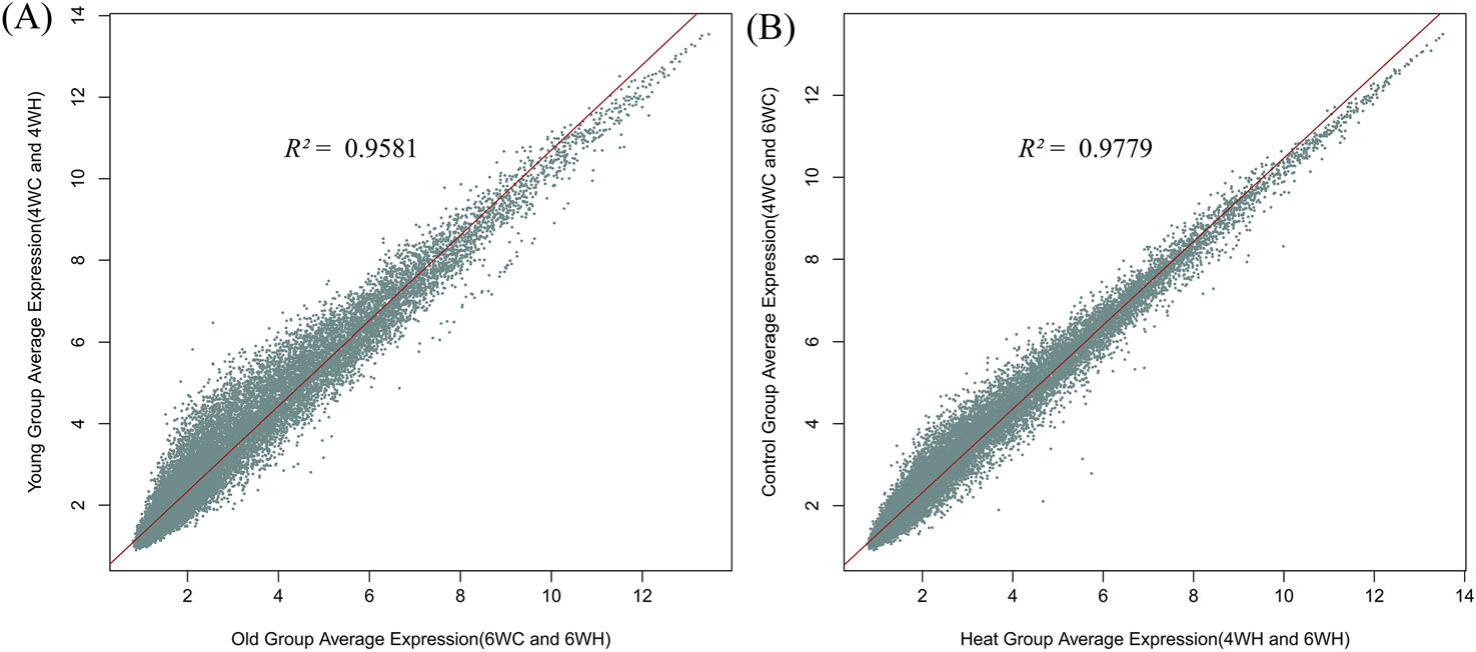
Correlation of average expression between experimental groups (A) Average expression comparison of the 4-week-old group (4WC and 4WH combined) against the 6-week-old group (6WC and 6WH combined). (B) Average expression comparison of the thermoneutral control group (4WC and 6WC combined) against high-temperature group (4WH and 6WH combined).

To further understand the similarity in genome-wide expression levels across experimental groups, we estimated the correlation coefficient of average expressions between different developmental stages (4WH and 4WC *versus* 6WH and 6WC, Figure 2. A.) and temperature levels (4WH and 6WH *versus* 4WC and 6WC, Figure 2. B.). The analysis suggested that the developmental stages caused slightly stronger fluctuations to the expression (*R^2^* = 0.96) than did the heat stress (*R^2 ^*= 0.98). The principal component and expression level correlation analyses together sufficiently indicated that the cellular and molecular response of breast muscle to developmental transition is more dynamic than to the hyperthermia condition.

### Comparison with RNA-sequencing data

**Figure 3.**
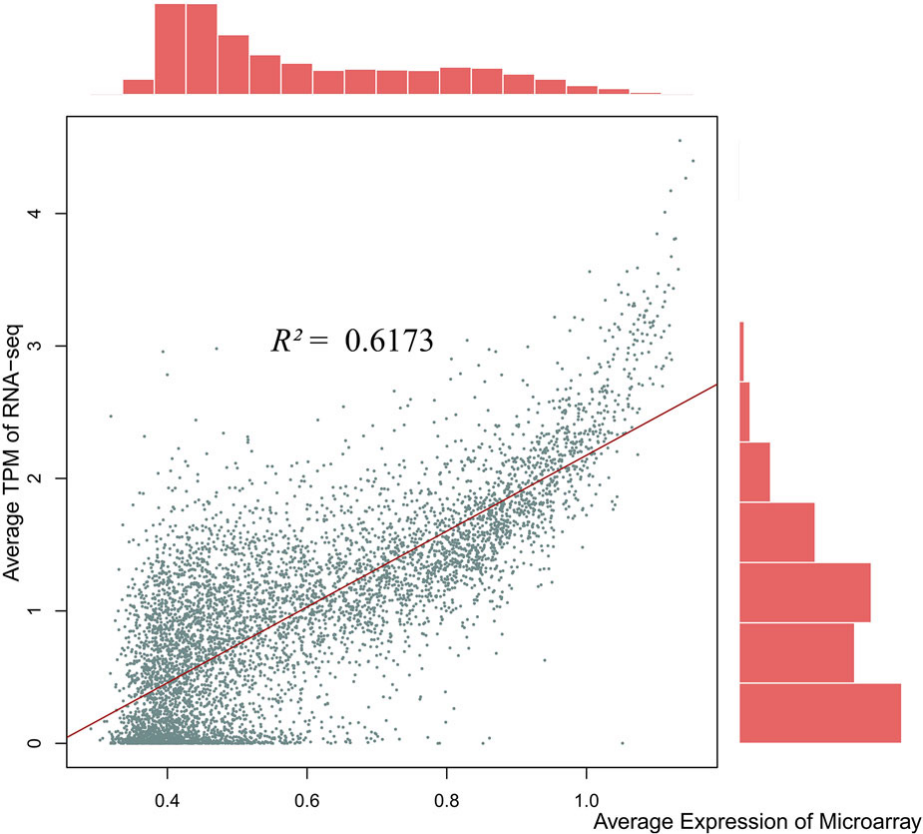
Concordance between different expression profiling platforms. Average TPM of RNA-seq against average expression across all groups from microarray in the same breast muscle tissue.

**Figure 4.**
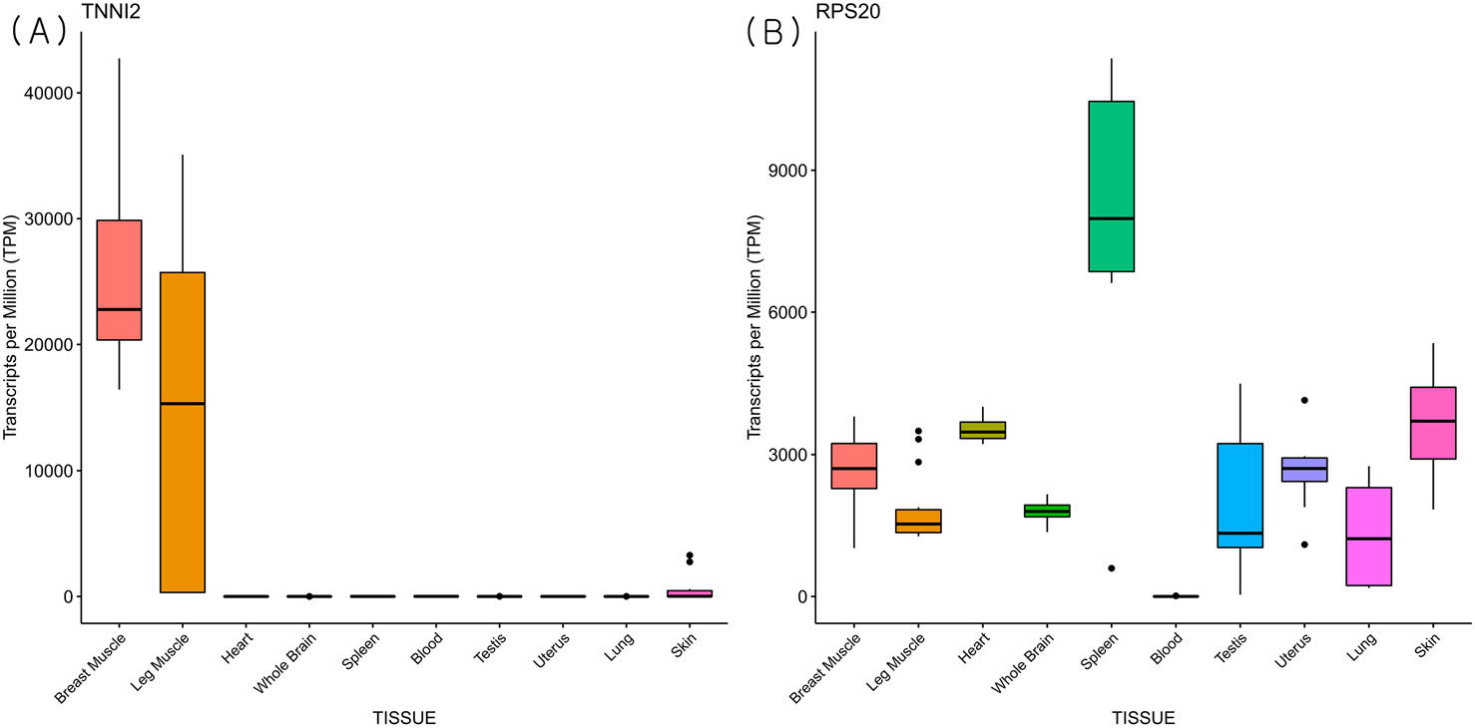
Boxplot of transcripts per million (TPM) for candidate genes across tissues from publicly available RNA-sequencing data. *TNNI2* (A) and *RPS20* (B) genes were selected from highly expressed genes across groups from microarray data.

To increase the reliability of our microarray data, the 17 publicly available RNA-seq datasets of the same breast muscle tissue were collected. The comparison with the RNA sequences of the different samples and conditions was made to estimate the overall cross-platform concordance of the genome-wide expression levels. We observed that the results obtained by RNA-seq and microarrays were highly reproducible (*R^2^* = 0.78). The result indicates that genes that show high expression from microarray, in general, tend to have high estimates in the common tissue of breast muscle (Figure 3). For example, the ten genes with the highest expressions across four experimental groups from microarray showed notably high TPM (transcripts per million) estimates in breast muscle, which is even more prominent when compared to other tissues (Figure 4, Supplementary Figure S1). The predated hybridization-based method such as microarray is criticized for detecting a limited range of expression and the lack of repeatability (Ioannidis et al. 2009; Byron et al. 2016). A high concordance estimate with a more recent platform implies the validity of quantitative measurement of expression in microarrays. It also indicates that specific treatment conditions may result in the differential expression on a subset of all available genes, however, in our study, the majority of expressions remained the same.

### The landscape of differential expression

**Figure 5.**
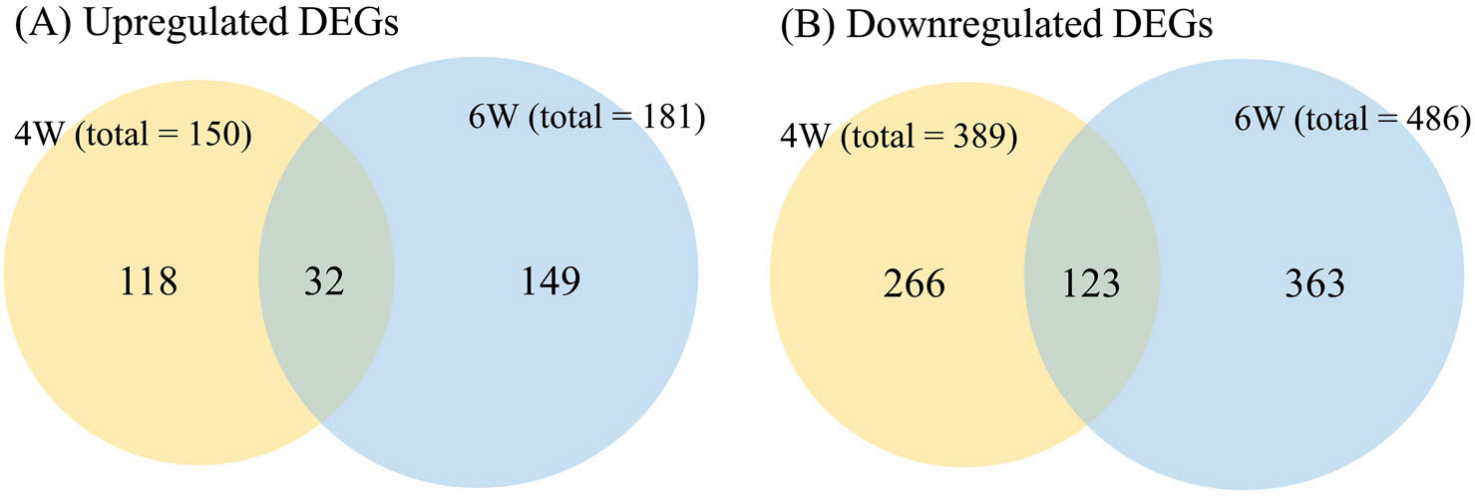
Summary of overall differentially expressed genes (DEGs) in 4-week (4W) and 6-week (6W) groups and common genes between experiments. The numbers represent genes significantly upregulated or downregulated relative to the thermoneutral group.

The transcriptome response of breast muscle was analyzed by measuring pairwise global gene expression changes between treatment and control groups at each developmental stage (4WH *versus* 4WC and 6WH *versus* 6WC). The ambient heat stress resulted in 539 and 667 DEGs in 4W and 6W respectively (Figure 5, Supplementary Table S2). A total of 115 DEGs were commonly differentially expressed in two developmental stages. In both growth phases, we noted that the transcriptome tended to be inhibitory rather than enhanced in response to heat (389 downregulated versus 150 upregulated DEGs and 486 downregulated versus 181 upregulated DEGs in 4W and 6W respectively). It is worth noting that we observed a higher number of DEGs in the later developmental stage (6W). The result is also supported by the significantly stronger overall absolute fold changes across the transcriptome in 6W compared to 4W (*P*< 2.2×10^−16^; Mann–Whitney *u* test).

### Significantly enriched pathways during hyperthermia

Analysis of Gene Ontology (GO) and KEGG pathway enrichment for DEGs of 4W indicated a significant overrepresentation for the categories associated with an immune response such as ‘antigen processing and presentation (GO: 0019882)’ and ‘antigen processing and presentation of peptide antigen via MHC class I (GO: 0002474)’ (Table 1, Supplementary Table S3). Cytokines and Toll-like receptors (TLRs) are essential markers for immunity, and TLRs especially have distinct roles of recognizing pathogen-associated molecular patterns and antigen presentation (Temperley et al. 2008). Myeloid cells are crucial effectors of the innate immune response and important regulators of adaptive immunity (Álvarez-Errico et al. 2015), and a corresponding biological process ‘negative regulation of myeloid cell differentiation (GO: 0045638)’ was significantly over-represented in 4W. The ubiquitination is a post-translational modification process ‘positive regulation of ubiquitin-protein transferase activity (GO: 0051443)’ that is crucial in the orchestration of an immune response by ensuring the proper functioning of key proteins within receptor signaling complexes that constitute the innate and adaptive immune systems (Pickart and Fushman 2004; Zinngrebe et al. 2014). During heat stress, the weight of lymphoid organs and thymus is known to decrease, which subsequently leads to a drastic dysfunction of T and B lymphocytes and ultimately reduces the antibody production (Zulkifli et al. 2000; Ghazi et al. 2012). These pathways together may explain the defense mechanisms, particularly the immune response under a high-temperature environment, which is governed by altered gene expression and regulation.

**Table 1.** Gene Set Enrichment Analysis of 4-week-chickens. The significant GO terms and KEGG pathways enriched from DEGs of the 4-week experiment. A list of genes in each term is provided in Supplementary Table S3.

Regulation	Pathways	Term	*P*-value
Upregulated	GO- Biological Pathways	antigen processing and presentation	1.08E-3
		atrial cardiac muscle cell development	2.05E-2
		reticulophagy	2.05E-2
		antigen processing and presentation of peptide antigen via MHC class I	2.05E-2
		regulation of membrane depolarization	4.07E-2
Downregulated	GO- Biological Pathways	mitotic nuclear division	1.09E-3
		response to muscle activity	4.66E-3
		negative regulation of myeloid cell differentiation	3.12E-2
		positive regulation of ubiquitin-protein transferase activity	3.12E-2
		protein neddylation	3.74E-2
		proteolysis	4.62E-02
	KEGG	Cell cycle	9.54E-04
		Oocyte meiosis	1.21E-2
		Fatty acid metabolism	3.43E-2

Heat stress apparently causes damage to cells by generating reactive oxygen species (ROS) (Lin et al. 2006; Goel et al. 2021), and associated pathways were significantly enriched in 6W (Table 2, Supplementary Table S3), the ‘reactive oxygen species metabolic process (GO: 0072593).’ The excessive production of ROS causes oxidative injury in the mitochondria of muscle cells which in turn decreases body weight gain (Quinteiro-Filho et al. 2010). Of the DEGs from both 4W and 6W, *PDK4* gene showed one of the highest fold changes, whose altered expression is responsible for abnormal mitochondria, ROS generation and cell death (Qian et al. 2004; Xu et al. 2018). As a defensive mechanism stimulated to protect the cell of damaged tissues and as an effort to restore the integrity of damaged tissues, genes associated with ‘positive response to wound healing (GO: 0090303)’ were differentially expressed under extremely high-temperature stress. One of the spontaneous responses to acute heat stress is increased expression of heat shock protein (HSP) genes that encode molecular chaperones associated with securing protein structures at high temperatures (Feder and Hofmann 1999), and although the corresponding biological processes were not significantly overrepresented, *HSP25* and *HSPB2* genes were downregulated in both growth phases. The concomitant decrease in expression may suggest an attenuation of the heat shock response from chronic heat stress.

**Table 2.** Gene Set Enrichment Analysis of 6-week-chickens. The significant GO terms and KEGG pathways enriched from DEGs of the 6-week experiment. A list of genes in each term is provided in Supplementary Table S3.

Regulation	Pathways	Term	*P*-value
Upregulated	GO- Biological Pathways	response to insulin	1.18E-3
		positive regulation of ossification	1.22E-3
		positive regulation of skeletal muscle fiber development	2.01E-3
		negative regulation of canonical Wnt signaling pathway	2.75E-3
		positive regulation of angiogenesis	4.96E-3
		positive regulation of fat cell differentiation	7.42E-3
		skeletal muscle cell differentiation	7.42E-3
		entrainment of circadian clock by photoperiod	8.64E-3
		circadian regulation of gene expression	9E-3
		positive regulation of myoblast differentiation	1.68E-2
		endothelial cell migration	1.92E-2
		negative regulation of G1/S transition of mitotic cell cycle	1.92E-2
		reactive oxygen species metabolic process	2.44E-2
		positive regulation of peptidyl-serine phosphorylation	2.54E-2
		positive regulation of transcription from RNA polymerase II promoter	2.75E-2
		positive regulation of cGMP metabolic process	2.87E-2
		negative regulation of glucocorticoid secretion	2.87E-2
		myotube differentiation involved in skeletal muscle regeneration	2.87E-2
		peptidyl-serine phosphorylation	3.43E-2
		canonical Wnt signaling pathway	04.09E-2
		Rho protein signal transduction	4.3E-2
	KEGG	Insulin resistance	2.71E-3
		FoxO signaling pathway	8.12E-3
Downregulated	GO- Biological Pathways	establishment of protein localization to plasma membrane	3.28E-3
		cholesterol biosynthetic process	1.09E-2
		negative regulation of epithelial cell proliferation	2.11E-2
		intracellular protein transport	2.68E-2
		nucleotide-excision repair	2.71E-2
		peptidyl-tyrosine dephosphorylation	3.13E-2
		positive regulation of wound healing	3.3E-2
		inositol phosphate dephosphorylation	3.3E-2
	KEGG	Tight junction	1.71E-2
		Steroid biosynthesis	2.05E-2
		Endocytosis	4.47E-2
		Synthesis and degradation of ketone bodies	4.64E-2

It is also noteworthy to mention that *CCK* gene was one of the most upregulated genes (*P*  = 4.54E-13, fold change = 15.09, Supplementary Table S2) in the 4W experiment. Although the tissue-specific expression is generally expected, preserved co-expression relationships between different tissue types have been reported; for example, genes expressed in brain tissue leave a transcriptional footprint in peripheral tissues (Cai et al. 2010). CCK is known to respond to heat stress by sending a signal to reduce the feed intake to the hypothalamus, resulting in dietary impairment in chickens (Richards 2003; Lei et al. 2013). In addition, it has been previously demonstrated that acute heat stress shifts the circadian clock (Buhr et al. 2010), by synchronizing clock genes to the heat stress cycle to adapt to cyclic heat stress and modulate the cell cycle and metabolism changes. The differential expression of *CCK* gene and circadian rhythm-related pathways ‘entrainment of circadian clock by photoperiod (GO: 0043153)’ and ‘circadian regulation of gene expression (GO: 0032922)’ in breast muscle may therefore reflect the surrogate for gene expression in the hypothalamus in response to thermal stress. Although the study intends to find the differential gene expression in breast muscle, the apparent alteration of expression in the brain may correlate the pattern in breast muscle, supported by the findings of moderately or highly correlated expression of brain and non-brain tissues pattern between in humans and mice (Davies et al. 2009; Aguet and Muñoz Aguirre 2017).

During thermal stress, as a response that helps minimize the production of metabolic heat, feed intake decreases, which can influence nutrient availability (Habashy et al. 2017). A strong ambient temperature negatively affects cell nutrient intake, and pathways putatively involved in the absorption of macronutrients in the intestine (Goel et al. 2021), ‘insulin response (GO: 0032868),’ ‘insulin resistance (hsa04931),’ and ‘fatty acid metabolism (hsa00071)’ were significantly enriched in 6W. Insulin increases glucose uptake in the muscle and fat, serving as the primary regulator of blood glucose concentration (Saltiel and Kahn 2001), and therefore insulin signaling and the regulation of glucose may be the key to nutrient uptake during heat stress and severe nutrient deficiency.

## Conclusion

Cells subjected to thermal stress respond by rewiring the transcriptome, including the differential expression of genes that govern the shift of rapid cell proliferation, growth processes, and metabolic adaptation (López-Maury et al. 2008). The significantly associated pathways in each 4W and 6W suggest that the transcriptomic responses of the rapidly growing broiler chickens to heat indicated differential cellular stress responses at different developmental stages. Changes in the transcriptome in 4W mainly indicated immune function, whereas chickens with higher body weight in the later stage (6W) responded to heat by altering the expression of genes encoding proteins that function in more diverse stress-associated pathways, including ROS clearance, cell nutrient intake, and shift in circadian rhythm. Our study is unique in assessing the transcriptional activity upon heat treatment at two contrasting growth phases in broiler chicken. The disproportionate ranges of enriched pathways at different ages may indicate that as the genome-wide expressions undergo precise and coordinated changes at defined stages of development, the transitions contribute to age-specific transcriptomic alteration in response to heat stress. These findings reveal that the chicken transcriptional response to heat is prompt, dynamic, and extensive, and may underlie the health outcomes observed during the thermal challenge.
